# A Bayesian approach to estimate the age-specific prevalence of *Schistosoma mansoni* and implications for schistosomiasis control

**DOI:** 10.1016/j.ijpara.2007.05.004

**Published:** 2007-11

**Authors:** Giovanna Raso, Penelope Vounatsou, Donald P. McManus, Eliézer K. N’Goran, Jürg Utzinger

**Affiliations:** a Molecular Parasitology Laboratory, Queensland Institute of Medical Research, Brisbane, Australia; b School of Population Health, The University of Queensland, Brisbane, Australia; c Department of Public Health and Epidemiology, Swiss Tropical Institute, Basel, Switzerland; d Unités de Formation et de Recherche des Biosciences, Université d’Abidjan-Cocody, Abidjan, Côte d’Ivoire; e Centre Suisse de Recherches Scientifiques, Abidjan, Côte d’Ivoire

**Keywords:** Age-prevalence prediction, Bayesian statistics, Immigration-death model, Kato-Katz technique, Schistosomiasis, *Schistosoma mansoni*

## Abstract

Models that accurately estimate the age-specific infection prevalence of *Schistosoma mansoni* can be useful for schistosomiasis control programmes, particularly with regard to whether mass drug administration or selected treatment should be employed. We developed a Bayesian formulation of an immigration-death model that has been previously proposed, which used maximum likelihood inference for estimating the age-specific *S. mansoni* prevalence in a dataset from Egypt. For comparative purposes, we first applied the Bayesian formulation of the immigration-death model to the dataset from Egypt. We further analysed data obtained from a cross-sectional parasitological survey that determined the infection prevalence of *S. mansoni* among 447 individuals in a village in Côte d’Ivoire. Three consecutive stool samples were collected from each participant and analysed by the Kato-Katz technique. In the Côte d’Ivoire study, the observed *S. mansoni* infection prevalence was 41.6% and varied with age. The immigration-death model was able to correctly predict 50% of the observed age group-specific point prevalences. The model presented here can be utilized to estimate *S. mansoni* community infection prevalences, which in turn helps in the strategic planning of schistosomiasis control.

## 1 Introduction

Schistosomiasis is a chronic and poverty-promoting disease caused by trematodes of the genus *Schistosoma*. The disease involves the gastro-intestinal and urinary-tracts of the human host. There are five schistosome species parasitizing humans, namely, *Schistosoma haematobium*, *Schistosoma intercalatum*, *Schistosoma japonicum*, *Schistosoma mansoni* and *Schistosoma mekongi* (Gryseels et al., 2006). Despite significant efforts to control schistosomiasis, the disease remains of considerable public health and economic importance in many developing countries. An estimated 207 million people are infected (Steinmann et al., 2006), and the global burden due to schistosomiasis is 4.5 million disability-adjusted life years (DALYs) lost (WHO, 2002) or even higher (King et al., 2005).

The Kato-Katz technique (Katz et al., 1972) is widely employed in epidemiological surveys focussing on intestinal schistosomiasis (de Vlas and Gryseels, 1992; Gryseels et al., 1994; Booth et al., 2003). This technique allows the identification of parasite eggs on thick smears prepared from faecal specimens that are examined under a light microscope. Yet this method lacks sensitivity and, therefore, a certain proportion of infected individuals remain undetected and hence community prevalence is under-estimated (de Vlas and Gryseels, 1992). Since light infections are particularly prone to be missed, repeated sampling of faecal specimens over multiple days has been recommended to increase diagnostic sensitivity (Utzinger et al., 2000).

A typical age-prevalence curve of *S. mansoni* infections in an endemic setting shows that the prevalence of infection increases from zero in newborns and normally reaches a peak in school-aged children, adolescents or young adults. In older age groups the prevalence decreases although it does not reach zero. Among other reasons, the curve is governed by exposure to infested freshwater bodies and immunological factors of the human host. In areas of intense transmission, the highest prevalence of infection is usually found among children aged 10–14 years, whereas a peak shift to adolescents and young adults is reported for areas of lower transmission with lower overall prevalences (Fulford et al., 1992; Woolhouse, 1998).

Holford and Hardy, in the mid-1970s, developed an immigration-death model to estimate an age-specific prevalence curve for schistosomiasis with data derived from a cross-sectional survey carried out in Egypt (Holford and Hardy, 1976). Their model considered the penetration of the parasite (i.e., cercariae) into the human host, the pairing of female and male worms within the host, the excretion of eggs by the host and the natural death of the parasite in the human host. However, this modelling approach entails several shortcomings. Firstly, not all of the model parameters can be estimated simultaneously due to numerical problems in finding maximum likelihood estimates and thus a parameter was constrained to a small set of fixed values. Secondly, no confidence intervals could be calculated for the predicted age-specific prevalences.

To remedy these shortcomings, we have now developed a Bayesian formulation of the above-mentioned immigration-death model and estimate the age-specific prevalence of *S. mansoni* infection. We compared the results with those obtained by Holford and Hardy (1976) by re-analysing the original dataset they had taken from Hairston (1965). Finally, we implemented the Bayesian model to our own data obtained from an endemic setting in Côte d’Ivoire and estimated the *S. mansoni* age-prevalence curve. Implications for schistosomiasis control are discussed.

## 2 Materials and methods

### 2.1 Study area and population

Details of the study areas and populations surveyed in Egypt (Hairston, 1965; Holford and Hardy, 1976) and Côte d’Ivoire (Raso et al., 2004b) have been presented before. With regard to the Côte d’Ivoire study, the data were obtained from a cross-sectional community-based survey, carried out in the village of Zouatta II in May 2002. This village is located 25 km east of the district town of Man in western Côte d’Ivoire.

### 2.2 Consent

In the Côte d’Ivoire study, a meeting was organized with the local authorities to ask for permission to work in their village. During the meeting, the aims and procedures of the study were explained. After consent was obtained the village authorities informed the community.

### 2.3 Field and laboratory procedures

Demographic data of village inhabitants (i.e., age and sex) were collected from 75 randomly selected households. Next, all members of the selected households were invited for repeated faecal examination (diagnosis of *S. mansoni*, soil-transmitted helminths and intestinal protozoa infections) and a single finger prick blood sample (diagnosis of malaria parasites) (Raso et al., 2004b). Here, we focus on *S. mansoni*. Each study participant was provided with a small plastic container the evening before the survey. The next morning, participants were invited to return the containers filled with a small amount of early morning stool. This procedure was repeated over three consecutive days, in order to increase the sensitivity of the diagnostic approach.

Faecal specimens were transferred to the laboratory in the town of Man. A single Kato-Katz thick smear, using 42 mg punched plastic templates, was prepared from each faecal specimen (Katz et al., 1972). After a clearing time of at least 30 min, the Kato-Katz thick smears were examined under a light microscope by experienced laboratory technicians. All *S. mansoni* eggs were counted. For quality control, 10% of the slides were re-examined by the senior laboratory technician.

### 2.4 Treatment

All individuals who were found *S. mansoni* egg-positive were treated with a single 40 mg/kg oral dose of praziquantel (WHO, 2002). Soil-transmitted helminth infections were treated orally with 400 mg albendazole. Adults who were neither infected with *S. mansoni* nor with soil-transmitted helminths received vitamins.

### 2.5 Statistical analysis

Analyses were performed in WinBUGS version 1.4 (Imperial College & Medical Research Council, London, UK). An individual was considered as positive for an infection with *S. mansoni* if at least one egg was found in at least one of the three Kato-Katz thick smear readings per person. The age of the study participants was stratified into 12 classes. To estimate the age-specific prevalence of *S. mansoni*, we applied a Bayesian formulation of the immigration-death model initially developed and described by Holford and Hardy (1976).

Model specifications were as follows: Let *X*_*j*_ and *P*_*j*_ be the number of infected individuals and the probability of infection with *S. mansoni* in age group *j*, respectively. We assumed that *X*_*j*_ arises from a binomial distribution *X*_*j*_ ∼ *Bin*(*P*_*j*_, *N*_*j*_) where *N*_*j*_ denotes the total number of individuals screened in that age group. We modelled *P*_*j*_ using the immigration-death model of Holford and Hardy (1976), which defines the infection prevalence as the probability of an individual to have at least one adult worm pair rather than a single worm, since *S. mansoni* eggs will only be observed in faeces when adult worms are present in pairs (male and female worms coupled). Based on the immigration model, *P*_*j*_ = [1 − exp(−*M*_*j*_)]^2^, which is determined under the assumption that the number of worms of either sex within an individual in the *j*th age category has a Poisson distribution with parameter *M*_*j*_. *M*_*j*_ depends on the worm immigration rate, *λ*_*j*_, which can be seen as the rate at which cercariae of either sex enter the host, and the worm death rate *δ*, which is the death of parasites within the host. It is also assumed that the rates at which worms immigrate and die are equal for worms of either sex. Further details about the derivation of *P*_*j*_ are given in the on-line Supplementary Data.

Holford and Hardy (1976) assumed that conditions in the transmission of *S. mansoni* remain constant over time, and hence *t* can be considered as one moment in time in a population that reflects what would be observed during a longitudinal study; thus this approach allows using data from cross-sectional surveys. An additional assumption in the previous model was that the contact rate of an individual with infected water (and thus exposure) decreases with age because one would expect that children play and swim in water more frequently than their adult counterparts. Therefore, Holford and Hardy (1976) consider a Gompertz type of function for *λ*_*j*_, which is an exponentially decreasing function (at a rate *B*) that reaches a lower plateau defined by *C*, that is *λ*_*j*_ = *A*[exp(−*Bt*_*j*_) + *C*], *A*, *B*, *C* > 0. *C* allows taking into consideration the case where some low water contact is maintained in older age groups; hence low transmission still occurs. It is also assumed that there is a constant worm death rate *δ*, irrespective of the age of the individual. Given these assumptions the expected number of worms of either sex present in an individual of age *t*_*j*_ is $M_{j}=\int_{0}^{t_{j}}\lambda_{j}(\tau )\exp(-\int_{\tau }^{t_{j}}\delta_{j}(x)dx)d\tau$, which is calculated from the immigration rate *λ*_*j*_ and the probability a worm is alive at *t*_*j*_ > *τ* given the worm has entered at *τ*. The last probability is quantified by the term $\exp(-\int_{\tau }^{t_{j}}\delta_{j}(x)dx)$ in the above equation of *M*_*j*_. Assuming a constant worm death rate *δ*_*j*_(*x*) = *δ*, the expected number of worms is given by $M_{j}=\exp(-\delta t_{j})\int_{0}^{t_{j}}\lambda_{j}(\tau )\exp(\delta \tau )d\tau$. Substituting *λ*_*j*_ by the above-mentioned Gompertz type form gives

$$M_{j}=\left\{\begin{array}{cc} A[t_{j}\exp(-\delta t_{j})+C^{\prime }\left(1-\exp(-\delta t_{j})\right)]\text{,} & B=\delta \\ A[\gamma^{-1}\left[\exp(-\delta t_{j})-\exp(-{\mathrm{Bt}}_{j})\right]+C^{\prime }(1-\exp(-\delta t_{j}))]\text{,} & B\neq \delta \end{array}\right)$$

where *γ* = *B* − *δ* and *C*′ = *C*/*δ*. When *γ* is equal to zero it forces the relative rate at which the immigration decreases with age *B* and the death rate *δ* of the worms to be the same. To avoid having zero in the denominator, it is necessary to formulate this di-equation for the case *B* = *δ*.

Following a Bayesian model specification, we adopted prior distributions for the model parameters. We compared different models with different sets of prior distributions such as normal priors with mean 0 and variances of either 1, 10 or 100, and gamma priors with mean 1 and variances of either 1, 10 or 100 for the parameters *A*, *C*′, *γ* and *δ*. In models with normal priors, *A*, *C*′ and *δ* were constrained to positive values. The deviance information criterion (DIC) was used for the appraisal of the best fitting model (Spiegelhalter et al., 2002). The DIC can have a negative or a positive value. A negative DIC can arise when the posterior distribution of a parameter is bimodal; in this case the posterior mean is a poor summary statistic and can give a very large deviance. Markov chain Monte Carlo (MCMC) simulation was employed to estimate the model parameters (Gelfand and Smith, 1990). We ran a single chain sampler with a burn-in of 10,000 iterations. Convergence was assessed by inspection of ergodic averages of model parameters and it was achieved before 100,000 iterations.

## 3 Results

### 3.1 Schistosoma mansoni age-prevalence curve in a dataset from Egypt

Table 1 presents a comparison of the estimated parameters *A*, *C*′, *δ* and *γ* from the original immigration-death model (Holford and Hardy, 1976) and our Bayesian-adapted model using different sets of priors. The Bayesian immigration-death model that best fitted the Egyptian data had a DIC of −2.45 and used gamma priors for *A*, *C*′ and *δ* and normal prior for *γ*. In this model, the posterior distribution of parameter *γ* had a bimodal shape, suggesting that *γ* arises from two different distributions. Holford and Hardy (1976) were not able to capture the bimodality of the *γ* parameter. It is worth mentioning that Holford and Hardy encountered a numerical problem for finding the maximum likelihood estimates when the *γ* parameter was added to the model. The authors therefore had to constrain *γ* to take on several values in order to find the maximum of the log likelihood for the model parameters *A*, *C*′ and *δ*. Subsequently, they estimated *γ* conditional on the estimates of the other parameters, using the maximum likelihood method. The Bayesian model we developed can estimate all parameters simultaneously.

**Fig. 1:**
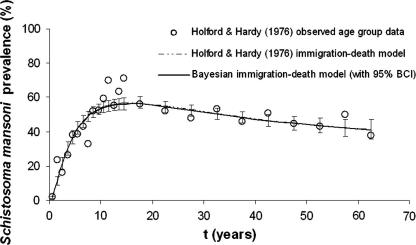
Observed and estimated age-prevalence for *Schistosoma mansoni* infections according to Holford and Hardy (1976), and according to a Bayesian approach, using the original data from Egypt. The figure has been adapted from Holford and Hardy (1976). Note that the age-prevalence curves obtained from the maximum likelihood approach and our best fitting Bayesian model are congruent.

**Fig. 2:**
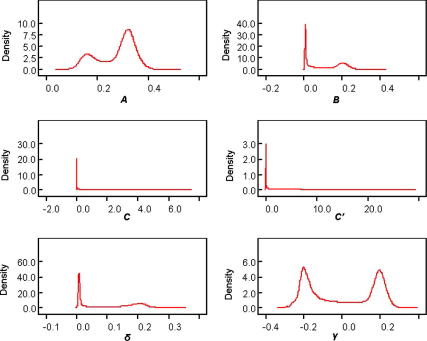
Posterior distributions of all model parameters according to the Bayesian approach, using original data from Egypt. The posterior distribution of several model parameters had a bimodal shape.

The predicted *S. mansoni* age-prevalences were identical for all three Bayesian models irrespective of the sets of priors utilized. Fig. 1 shows that the *S. mansoni* age-prevalence curves obtained from the maximum likelihood approach and our best fitting Bayesian model are congruent. Note that the Bayesian approach allowed determining Bayesian credible intervals (BCI), which proved to be very narrow. The standard deviation (SD) for the predicted age group prevalences ranged from 0.31% to 3.1%. A total of 14 (56%) out of 25 observed age-specific prevalences fell within the 95% BCI of the posterior mean inferred from the Bayesian immigration-death model. The posterior distributions of all model parameters from the best fitting model are shown in Fig. 2.

### 3.2 Schistosoma mansoni age-prevalence curve in a dataset from Côte d’Ivoire

#### 3.2.1 Population sample

The Côte d’Ivoire study enrolled 561 individuals, aged between 5 days and 91 years (Raso et al., 2004a,b). Overall, 447 individuals (79.7%) provided three consecutive stool specimens. Further analyses presented here focussed on this cohort. There were 235 males (52.6%) and 212 females (47.4%).

#### 3.2.2 Observed age-specific prevalence of S. mansoni

**Fig. 3:**
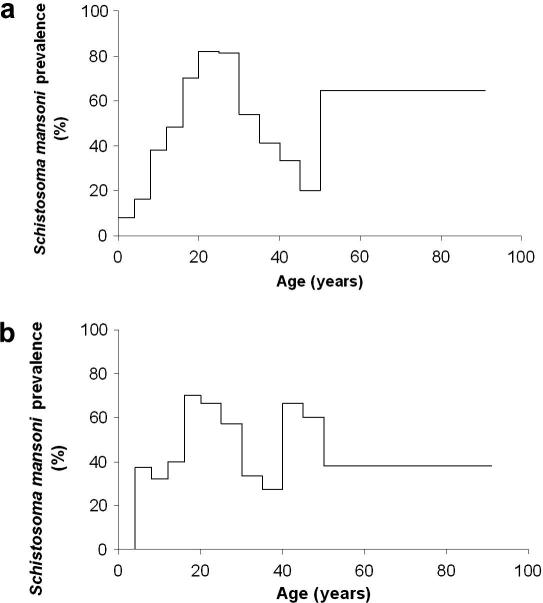
Observed age group prevalence of *Schistosoma mansoni* infections, stratified by sex (a: males, b: females), among 447 study participants from a community of western Côte d’Ivoire. The results are based on three Kato-Katz thick smear readings. The first and highest peak prevalence was observed in 15–19 year-old females. Males had their first peak prevalence in their 20s. Among females there was a second peak in the 45–49 years age group, whereas males showed a second peak at ⩾50 years.

The overall *S. mansoni* infection prevalence in the village of Zouatta II, based on three Kato-Katz thick smear readings from each individual, was 41.6% (Table 2). Fig. 3 shows that the age-specific prevalence ranged from 3.9% (in the youngest age group) to 72.4% (observed in 20–24 year-old participants). The second highest infection prevalence was observed in individuals aged 16–19 and 25–29 years. There was a tendency for a decrease in prevalence among subjects aged 30 years and above, but two small peaks were observed at ages 40–44 years, as well as 50 years and above. These two small peaks in the older age groups became clear when stratifying the prevalence curves by sex. Among females there was a second peak in the 45–49 years age group, whereas males showed a second peak at ⩾50 years.

Table 2 also summarizes the results based on only one or two, rather than all three, Kato-Katz thick smears. These data confirm that the rate of detecting *S. mansoni*-positive cases increased substantially with an increased sampling effort (from 25.3% with a single Kato-Katz thick smear to 41.6% following three Kato-Katz thick smear readings).

#### 3.2.3 Bayesian immigration-death model

Table 3 displays the mean estimates inferred from the Bayesian immigration-death model using three different sets of prior distributions. The best fitting model had a DIC of −2.74 and normal priors for all four parameters *A*, *C*′, *δ* and *γ*.

**Fig. 4:**
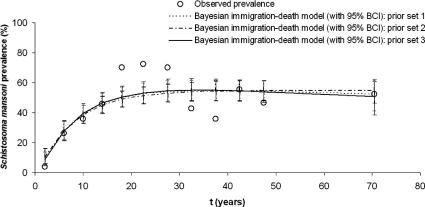
Comparison of observed *Schistosoma mansoni* age-prevalence data with the age-specific prevalence curve (including age group specific 95% Bayesian credible intervals (BCIs)) as inferred from the Bayesian immigration-death model using three different prior sets for the community of western Côte d’Ivoire. The 95% BCIs were slightly wider with this dataset compared with the original dataset from Egypt (Holford and Hardy, 1976).

**Fig. 5:**
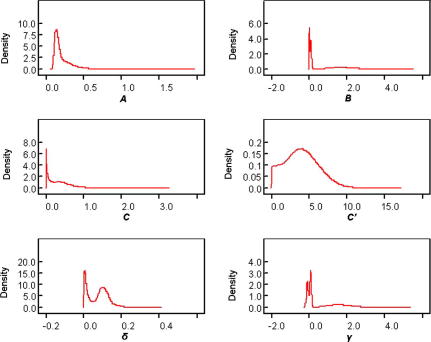
Posterior distributions of all model parameters according to the Bayesian approach, using data from the Côte d’Ivoire study. The posterior distribution of model parameters *B*, *δ* and *γ* had a bimodal shape.

Fig. 4 shows the observed *S. mansoni* age-prevalence data, as well as the age-specific prevalence curve (including age group specific 95% BCIs) as inferred from the Bayesian immigration-death model using three different prior sets for the community of western Côte d’Ivoire. The 95% BCIs were slightly wider with this dataset compared with the Egyptian dataset (Holford and Hardy, 1976). The SD of the predicted age group prevalences ranged between 3.2% and 5.8%. A total of six (50%) out of 12 observed age-specific prevalences fell within the 95% BCI of the posterior mean inferred from the best fitting Bayesian immigration-death model. The posterior distribution of the parameter *γ* had a bimodal shape, which was also found with the Egypt data. The posterior distributions of *γ* and other model parameters are shown in Fig. 5.

**Fig. 6:**
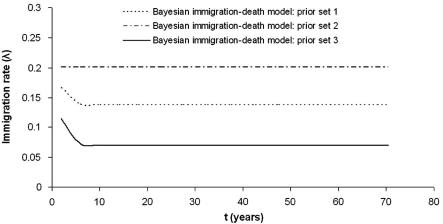
Immigration rate (*λ*_*j*_) curves for the models with different prior sets 1, 2 and 3 for the Côte d’Ivoire data. The model with prior set 1 estimates the immigration rate almost as constant, hence there is little dependence with time (age). The immigration rate curves for the models with prior sets 2 and 3 (best fitting model) show that the immigration rates in the youngest age groups are higher and decrease rapidly to an asymptote.

Fig. 6 displays the immigration rate curves for the model, using three different prior sets. It appears that the model with prior set 1 estimates the immigration rate *λ*_*j*_ almost as constant. Thus in the model with this prior set the immigration rate has little dependence with *t* (age). The immigration rate curves for the models with prior sets 2 and 3 (best fitting model) show that the immigration rates in the youngest age groups are higher and decrease rapidly to an asymptote.

## 4 Discussion

For the optimal design of schistosomiasis control it is essential to gain baseline information on the prevalence, infection intensity and/or morbidity at the community level in order to select the appropriate control strategy. The World Health Organization (WHO) recommends mass drug administration in communities where schistosome infection prevalences among school-aged children are equal or higher than 50% (WHO, 2002). If the prevalence among school-aged children are moderate (⩾10% but below 50%) or low (<10%), mass treatment is only recommended for school-aged children (WHO, 2002). In this case, treatment is targeted only to enrolled and non-enrolled children in schools with moderate prevalences every 2 years and in schools with low prevalences only twice; first at school entry and second before leaving school.

However, in countries where resources are constraint, it can be difficult to gather accurate baseline information, which is necessary for strategic planning as detailed above. In such a case, models that estimate accurate age-prevalence curves can help in the choice of appropriate control interventions for communities based on a sub-sample of results obtained from parasitological examinations. Once such models have been established, it is conceivable that a health worker could input results from a parasitological examination undertaken on a sub-sample of a population into a computer and receive an estimation of the community age-prevalence as graphical output through the use of nomograms, which are related to the models.

The aim of this study was to estimate the age-specific prevalence curve of *S. mansoni* in a Côte d’Ivoire community, utilizing an immigration-death model initially developed by Holford and Hardy (1976) some 30 years ago, and to further adapt their model by a Bayesian-based statistical approach. As expected, the lowest *S. mansoni* infection prevalence was observed in infants and very young children. The prevalence increased to over 70% in adolescents and young adults (aged 16–29 years), most likely as a result of frequent exposure to infested water by individuals in these age groups (Babiker et al., 1985; Chandiwana and Woolhouse, 1991; Kloos et al., 1998). In adults, the prevalence decreased with age. The two most likely explanations for this decrease in schistosome prevalence with age normally observed in adolescence or young adulthood that have been cited in the literature are as follows. Firstly, there is a decrease in exposure to infested water. Secondly, previous exposure leads to the acquirement of some protective immunity (Woolhouse et al., 1991; Woolhouse, 1998). In the current study, the peak in the prevalence of *S. mansoni* was reached in older age groups than expected, which might be due to the moderate infection prevalence and transmission in this setting. Experimental and field studies have shown that the prevalence peak is reached in younger age groups if transmission is high, whereas if transmission is low-to-moderate, the prevalence peak is reached at a later age. This pattern has been described in the literature as ‘peak shift’ (Fulford et al., 1992; Woolhouse, 1998).

By employing a Bayesian approach, we were able to overcome some of the inherent problems that Holford and Hardy faced in the mid-1970s, most notably the estimation of all model parameters. In contrast to their frequentist approach, our Bayesian model specification via MCMC algorithms offers the flexibility to fit a rather complex model and to obtain estimates for the whole distribution of the unknown parameters, including point and interval estimates (Basáñez et al., 2004). In contrast, the frequentist approach often only gives estimates and crude standard errors based on asymptotic results (Basáñez et al., 2004). Furthermore, our approach allowed capture of the bimodal distribution of the *γ* parameter, an important feature that Holford and Hardy (1976) failed to observe. Finally, the Bayesian approach facilitated comparison of model outcomes based on different prior sets, and hence improves our understanding of the mechanics of the model.

Although it is difficult to interpret the bimodal shape of *γ*, it is conceivable that it might be directly related to the bimodal nature of the age-prevalence curve. The Côte d’Ivoire data clearly show a bimodal age-prevalence curve; this seems to be also true for the Egyptian dataset, although much less pronounced. Since there is this bimodality of the age-prevalence, it could also mean that the relative rate at which the immigration rate decreases with age (*B*) and/or the rate at which the worms in the host die naturally (*δ*), arises from a mixture of distributions, since *γ* = *B* − *δ*. In fact similarly to *γ*, the posterior distributions of *B* and *δ* had a bimodal shape for the Côte d’Ivoire data. This bimodality could be related to patters of water contact and, as emphasized later in the discussion, might also explain the bimodal shape found for the age-prevalence curve.

Although the Kato-Katz technique is an inexpensive, relatively rapid and quantitative diagnostic technique, a major shortcoming is its low sensitivity, particularly in areas where *S. mansoni* infection intensities are low (Engels et al., 1996; Utzinger et al., 2001). The low sensitivity arises from day-to-day variation and inter-specimen variations of egg output (Engels et al., 1996, 1997; Utzinger et al., 2001). By increasing the sampling effort we could improve the sensitivity but the prevalence is still likely to be under-estimated. Previous studies have employed Bayesian methods to estimate ‘true’ prevalences for helminth infections, examples being porcine cysticercosis, onchocerciasis, strongyloidiasis and schistosomiasis japonica (Joseph et al., 1995; Carabin et al., 2003; Dorny et al., 2004; Wang et al., 2006). The sensitivity of the Kato-Katz technique is positively correlated to the infection intensity; the higher the number of eggs excreted in faeces, the lower the false negative rates (de Vlas and Gryseels, 1992; Booth et al., 2003; Raso et al., 2004a). In a recent study, Carabin and colleagues (2005) analysed *S. japonicum* infection intensity data obtained from a cross-sectional study carried out in The Philippines, by utilizing a Bayesian cumulative-logit model. The authors are to be applauded, as they accounted for an intensity-dependent sensitivity of the diagnostic technique. We hypothesise that it will be important to integrate a similar approach when modelling age-prevalence patterns of infectious disease data.

The immigration-death model by Holford and Hardy (1976) assumes that water contact decreases constantly to a non-zero level in older age groups, which is controlled by the Gompertz function. Interestingly, we found a second peak in prevalence both in men and women in the Côte d’Ivoire study, which has been described before in age-intensity curves (Mutapi et al., 2003). We consider two likely causes for this observation. Firstly, some immunological factors might change with advanced age; hence older individuals are less protected against schistosome challenges (Naus et al., 2003). Second, the second peak might be caused by an increase of water contact resulting from a change in occupational activities at a later age. It appears that the immigration-death model by Holford and Hardy (1976) is less suited to the Côte d’Ivoire data, compared with the Egyptian data. Our validation, which compares the observed age group prevalence with the posterior distribution of the 95% BCIs, showed that only 50% of the age group prevalence was correctly predicted with the Côte d’Ivoire data, compared with 56% with the Egyptian data. The model under-estimates the peak prevalence of adolescence and young adulthood and overestimates the burden for persons in their 30s. However, also with the Egyptian data, the peak prevalence was under-estimated. Future work should concentrate on developing a model that also captures variation in exposure to infested water in older age groups. This might be done by modifying the Gompertz function or by replacing the Gompertz function with a mixture of distributions. Furthermore, it would be interesting to develop hybrid models by introducing an immunity function as presented by Crombie and Anderson (1985).

In conclusion, our approach utilizing Bayesian statistics to estimate the age-prevalence of *S. mansoni* offers the possibility of flexible modelling via MCMC algorithms and, therefore, presents a computational advantage, which in our case allowed drawing inference for all parameters of the immigration-death model. Future work should focus on further adapting the models to changes in exposure in older age groups and to take into account intensity-dependent sensitivity. The Bayesian immigration-death model can become an important tool for planning control strategies in western Côte d’Ivoire and elsewhere in sub-Saharan Africa where schistosomiasis is regarded as a neglected tropical disease (Hotez et al., 2006), although it remains of considerable public health and economic significance.

## Figures and Tables

**Table 1:** Comparison of parameter estimates using model specifications according to Holford and Hardy (1976) or a Bayesian approach with different sets of prior distributions

Parameter	Holford and Hardy (1976)		Bayesian approach						
			Prior set 1: *A*, *C*′ ∼ N(0,100)I(0,)^b^, *γ* ∼ N(0,100) and *δ* ∼ N(0,1)I(0,)		Prior set 2: *A*, *C*′ ∼ N(0,10)I(0,), *γ* ∼ N(0,10) and *δ* ∼ N(0,1)I(0,)		Prior set 3: *A*, *C*′ ∼ Ga(0.01,0.01)^c^, *γ* ∼ N(0,10) and *δ* ∼ Ga(1,1)		
	Maximum likelihood estimate	SD	Mean estimate from posterior distribution	SD	Mean estimate from posterior distribution	SD	Mean estimate from posterior distribution	SD	
*A*	0.2842	0.0904	0.2077	0.0668	0.2472	0.0633	0.2733	0.0756	
*C*′	3.1644	2.5509	5.019	2.3800	3.4680	1.8320	2.248	2.777	
*δ*	0.0338	0.0552	0.1218	0.0696	0.1004	0.0765	0.1116	0.0889	
*γ*	0.1468	0.1158	−0.0169	0.1349	0.0214	0.1543	−0.0023	0.1767	
									
DIC^a^			156.29		132.02		−2.45		

a DIC, deviance information criterion; measures the model fit, the smaller the value the better the model fits the data.

b N(0,100)I(0,) indicates a Normal distribution with mean 0 and variance 100, constrained to positive values.

c Ga(0.01,0.01) indicates a Gamma distribution with mean 1 and variance 100.

**Table 2:** Number of people examined, number and percentage of people infected with *Schistosoma mansoni* in the village of Zouatta II, western Côte d’Ivoire, stratified by 12 age groups, in relation to different diagnostic efforts (1, 2 or 3 Kato-Katz thick smears)

Age group (years)	*t*_*j*_	No. of people examined	3 Kato-Katz thick smears		2 Kato-Katz thick smears		1 Kato-Katz thick smear	
			*S. mansoni* positive	*S. mansoni* prevalence (%)	*S. mansoni* positive	*S. mansoni* prevalence (%)	*S. mansoni* positive	*S. mansoni* prevalence (%)
<4	2	51	2	3.9	1	2.0	0	0.0
4–7	6	69	18	26.1	13	18.8	6	8.7
8–11	10	59	21	35.6	18	30.5	13	22.0
12–15	14	37	17	46.0	16	43.2	11	29.7
16–19	18	20	14	70.0	12	60.0	9	45.0
20–24	22.5	29	21	72.4	20	69.0	17	58.6
25–29	27.5	30	21	70.0	20	66.7	16	53.3
30–34	32.5	28	12	42.9	10	35.7	8	28.6
35–39	37.5	28	10	35.7	8	28.6	4	14.3
40–44	42.5	18	10	55.6	9	50.0	5	27.8
45–49	47.5	15	7	46.7	5	33.3	3	20.0
⩾50	70.5	63	33	52.4	28	44.4	21	33.3
								
Total		447	186	41.6	160	35.8	113	25.3

**Table 3:** Results from a Bayesian immigration-death model using three different sets of priors for estimation of *Schistosoma mansoni* age-specific prevalence among community members in the village of Zouatta II, western Côte d’Ivoire

Parameter	Bayesian model with prior set 1: *A*, *C*′ ∼ N(0,100)I(0,)^b^, *γ* ∼ N(0,1) and *δ* ∼ N(0,1)I(0,)		Bayesian model with prior set 2: *A*, *C*′ ∼ N(0,10)I(0,), *γ* ∼ N(0,10) and *δ* ∼ N(0,10)I(0,)		Bayesian model with prior set 1: *A*, *C*′ ∼ N(0,10)I(0,), *γ* ∼ N(0,1) and *δ* ∼ N(0,1)I(0,)	
	Mean estimate of the posterior distribution	SD	Mean estimate of the posterior distribution	SD	Mean estimate of the posterior distribution	SD
*A*	0.1310	0.0659	0.4759	0.2941	0.2187	0.1180
*C*′	10.700	5.2920	3.5450	1.7470	4.1060	2.2240
*δ*	0.0908	0.0428	0.1177	0.0305	0.0725	0.0507
*γ*	0.6604	0.7613	4.2540	2.1920	0.7255	0.9156
						
DIC^a^	70.76		57.73		−2.74	

a DIC, deviance information criterion; measures the model fit, the smaller the value the better the model fits the data.

b N(0,100)I(0,) indicates a Normal distribution with mean 0 and variance 100, constrained to positive values.
