# Supplementary material for: A Bayesian approach to estimate the age-specific prevalence of Schistosoma mansoni and implications for schistosomiasis control
Source: Int J Parasitol. 2007 Nov;37(13-3):1491–500. doi: 10.1016/j.ijpara.2007.05.004 (PMC2756495; doi:10.1016/j.ijpara.2007.05.004)
Supplement: Supplementary file 1 — Supplementary data [file mmc1.doc]

**Supplementary Data**

The number of worms $N_{i}(t_{j})$ of either sex *i* (*i* = 1,2) that an individual of age $t_{j}$ harbours follows a Poisson distribution with parameter $M_{j}$. An individual is considered positive for a *Schistosoma mansoni* infection only when eggs of the parasite are found in faecal samples, which requires the presence of adult worm pairs in an individual. Let $k(t_{j})$ be the number of worm pairs harboured in an individual, then . Holford and Hardy (1976) defined the prevalence as the probability of an individual to be infected with at least one worm pair, that is where derives from the following Poisson distribution: .
